# Development and validation of PAMPA-BBB QSAR model to predict brain penetration potential of novel drug candidates

**DOI:** 10.3389/fphar.2023.1291246

**Published:** 2023-12-01

**Authors:** Rintaro Kato, Wenyu Zeng, Vishal B. Siramshetty, Jordan Williams, Md Kabir, Natalie Hagen, Elias C. Padilha, Amy Q. Wang, Ewy A. Mathé, Xin Xu, Pranav Shah

**Affiliations:** ^1^ National Center for Advancing Translational Sciences (NCATS), 9800 Medical Center Drive, Rockville, MD, United States

**Keywords:** parallel artificial membrane permeability assay (PAMPA), blood-brain barrier (BBB), drug discovery, quantitative structure-activity relationship (QSAR), computational drug design, ADME

## Abstract

Efficiently circumventing the blood-brain barrier (BBB) poses a major hurdle in the development of drugs that target the central nervous system. Although there are several methods to determine BBB permeability of small molecules, the Parallel Artificial Membrane Permeability Assay (PAMPA) is one of the most common assays in drug discovery due to its robust and high-throughput nature. Drug discovery is a long and costly venture, thus, any advances to streamline this process are beneficial. In this study, ∼2,000 compounds from over 60 NCATS projects were screened in the PAMPA-BBB assay to develop a quantitative structure-activity relationship model to predict BBB permeability of small molecules. After analyzing both state-of-the-art and latest machine learning methods, we found that random forest based on RDKit descriptors as additional features provided the best training balanced accuracy (0.70 ± 0.015) and a message-passing variant of graph convolutional neural network that uses RDKit descriptors provided the highest balanced accuracy (0.72) on a prospective validation set. Finally, we correlated *in vitro* PAMPA-BBB data with *in vivo* brain permeation data in rodents to observe a categorical correlation of 77%, suggesting that models developed using data from PAMPA-BBB can forecast *in vivo* brain permeability. Given that majority of prior research has relied on *in vitro* or *in vivo* data for assessing BBB permeability, our model, developed using the largest PAMPA-BBB dataset to date, offers an orthogonal means to estimate BBB permeability of small molecules. We deposited a subset of our data into PubChem bioassay database (AID: 1845228) and deployed the best performing model on the NCATS Open Data ADME portal (https://opendata.ncats.nih.gov/adme/). These initiatives were undertaken with the aim of providing valuable resources for the drug discovery community.

## 1 Introduction

The brain contains a highly restrictive interface known as the blood-brain barrier (BBB) (Alahmari, 2021). The BBB comprises of endothelial cells which form tight junctions inhibiting the passage of certain molecules to provide optimal central nervous system (CNS) functioning (Dotiwala et al., 2023). One of the major hurdles in CNS drug discovery is developing a BBB-penetrant drug (Pardridge, 2005; Pardridge, 2012; Khawli and Prabhu, 2013). High passive BBB permeability is beneficial for CNS drug candidates, enabling rapid establishment of distribution equilibrium between plasma and brain (Di et al., 2013). In addition to playing a critical role for brain penetration, passive permeability is translatable across tissues and different species including humans (Di et al., 2020).

BBB permeability can be assessed in three general ways; *in vivo* in lab animals, cell based *in vitro*, and non-cell based *in vitro* assays. Some common *in vitro* assays include Madin-Darby Canine Kidney cells (MDCK-MDR1), human colon adenocarcinoma derived Caco-2 cells, and Organ-on-chip assay. Both MDCK-MDR1 and Caco-2 cells form confluent monolayers that model active and passive transport (Volpe, 2011). Organ-on-chip is an innovative assay that involves the use of microfluidic chips containing miniature tissues that mimic the structure and function of natural organs (Leung et al., 2022). Utilizing induced pluripotent stem cell (iPSC)-derived brain microvascular endothelial-like cells (iBMECs), the human BBB-Chip replicates marker-specific brain vasculature and physiologically relevant transendothelial electrical resistance. This platform effectively predicts BBB-permeability of pharmacological compounds (Vatine et al., 2019). Although routinely used for assessing brain penetration, these assays are laborious, time-consuming, low-to-moderate throughput, expensive and several aspects of these assays are not amenable to automation. A popular non-cellular *in vitro* assay used to assess permeability is the Parallel Artificial Membrane Permeability Assay (PAMPA). This assay is simple, low cost, high-throughput, and the entire assay from start to finish is amenable to automation. Due to its non-cellular nature, this assay cannot assess active efflux transport however, this is offset by the fact that majority of CNS drugs are passively diffused (Banks, 2009; Mikitsh and Chacko, 2014). Furthermore, PAMPA’s adaptability to measuring permeability across different membranes such as BBB, gastrointestinal tract (GIT) and skin, makes it an exceptional screening tool especially in early drug discovery.

During preclinical discovery, many compounds, often in the thousands, are screened for their potential as drug candidates. However, it is estimated that only about 10 out of every 1,000 screened compounds become optimized leads, which then proceed to preclinical *in vivo* testing (Markossian et al., 2004; Hughes et al., 2011; Siramshetty et al., 2021). This highlights the rigorous and selective nature of the drug discovery process, where only a small fraction of initial compounds show promise for further development. Studies have estimated that ∼90% of drug candidates fail after entering phase I clinical trials, indicating the significant challenges involved in drug development (Sun et al., 2022). Moreover, the failure rate tends to be even higher for drugs targeting the CNS (Morofuji and Nakagawa, 2020). Due to the high attrition rates and escalating costs associated with drug discovery, there is a growing need to streamline and optimize the drug discovery process. Quantitative structure-activity relationships (QSAR) combined with machine learning approaches, have been successfully employed in multiple stages of the drug discovery process (Muratov et al., 2020; Shah et al., 2020; Siramshetty et al., 2020; Gonzalez et al., 2021; Kabir et al., 2022; Williams et al., 2022).

In this study, we aim to develop and optimize a robust QSAR model that can accurately predict BBB permeability using experimental PAMPA-BBB data. The PAMPA-BBB model was developed using a diverse dataset of ∼2,000 compounds representing >60 small molecule drug discovery projects at the National Center for Advancing Translational Sciences (NCATS). We evaluated both classical and advanced machine learning techniques to develop prediction models and the best predictive model with training and validation accuracies over 70% was made publicly accessible on the NCATS Open Data ADME portal (https://opendata.ncats.nih.gov/adme/). Additionally, we found a 77% categorical correlation between *in vitro* PAMPA-BBB data and *in vivo* brain/plasma (B/P) ratios demonstrating the value of the PAMPA-BBB assay as a rapid rank ordering tool for novel discovery compounds.

## 2 Materials and methods

### 2.1 Materials

Dimethyl sulfoxide (DMSO, high performance liquid chromatography (HPLC) grade), caffeine, progesterone, and carbamazepine were purchased from Sigma-Aldrich (St. Louis, MO). Brain sink buffer (Catalog #110674), BBB-1 lipid solution (Catalog #110672), 96-well stirwell sandwich plates (Catalog #110243), and preloaded support plate [for use with 96-well stirwell sandwich plate (Catalog #120551-Supp)] were purchased from Pion Inc. (Billerica, MA). UV plates (Catalog #675801) were purchased from Greiner BIO-ONE (Monroe, NC). 0.5 M potassium phosphate buffer solution, pH 7.4 (Catalog #J61413) was purchased from Thermo Fisher Scientific (Waltham, MA).

### 2.2 PAMPA-BBB permeability assay

The stirring Double-Sink™ PAMPA-BBB method patented by Pion Inc. (Billerica, MA) was employed to determine the permeability of compounds (Mandic, 2013). The PAMPA lipid membrane, which consists of porcine brain lipid extract dissolved in alkane (Pion Inc.), was optimized to predict BBB passive permeability. This membrane was immobilized on a PVDF matrix of a 96 well “acceptor” filter plate placed on top of a 96 well “donor” plate containing coated magnetic stirrers. The test articles, stocked in 10 mM DMSO solutions, were diluted to 0.05 mM in aqueous phosphate buffer and the concentration of DMSO was 0.5% in the final solution. During the 60-min permeation study period, conducted at room temperature, the test samples in the donor compartment were stirred using the Gutbox technology (Pion Inc.) to reduce the aqueous boundary layer to 60 µm. The test article concentrations in the “donor” and “acceptor” compartments were measured using a UV plate reader (Nano Quant, Infinite^®^ 200 PRO, Tecan Inc., Männedorf, Switzerland). Permeability (P_e_) calculations were performed using Pion Inc. Software and were expressed in units of 10^−6^ cm/s.

### 2.3 Permeability data

Data for more than 2,000 compounds were generated; however, after standardization and removal of duplicates, the final dataset consisted of 1,958 unique compounds. Among them, 1,794 were considered as internal compounds and were used to train the machine learning models. For the purpose of internal validation, the training set was further divided into internal training set and internal test set that comprise 80% and 20% of the training set compounds respectively. An independent set of 164 compounds were treated as the validation set to validate the trained models. An overview of the training and validation compound sets is provided in Table 1. A permeability cutoff value of 10 × 10^−6^ cm/s was employed to assign binary class labels: compounds with a permeability value less than or equal to 10 × 10^−6^ cm/s were categorized as class 1 or “low permeability” compounds, while compounds with a permeability value greater than 10 × 10^−6^ cm/s were designated as class 0 or “moderate to high permeability” compounds. Our study deals with a significantly imbalanced dataset, with roughly 70% of the training data belonging to class 0.

**TABLE 1:** Summary of datasets employed for model development in this study.

	Internal training set		Validation set	
Class distribution	Class 1	Class 0	Class 1	Class 0
	554	1,240	33	131
Total	1,794		164	

### 2.4 Molecular descriptors

Molecular descriptors derived from chemical structures were employed as features when building QSAR models. Descriptors were generated using two different software in this study. We employed the RDKit toolkit to create a comprehensive set of 212 descriptors. Subsequently, descriptors with over 15% missing values and those exhibiting constant values were eliminated. After filling any data gaps with the corresponding column’s mean value, the final descriptor count was 197. These descriptors describe different properties of a molecule as an array of real values rather than directly encoding chemical structure information. Molecular fingerprints on the other hand encode chemical structure information in a bit string where each bit corresponds to a substructure. Again, RDKit toolkit was used to generate Morgan fingerprints with a radius of 2 and a total of 1,024 bits per fingerprint. RDKit’s Morgan fingerprint (radius = 2) is equivalent to the extended connectivity fingerprint ECFP4 and both implementations have been popularly employed in drug discovery tasks such as virtual screening and target activity predictions. These fingerprints represent the presence of specific circular substructure features around individual atoms in a molecule. Molecular Operating Environment (MOE) software from Chemical Computing Group197 was employed to calculate 2D descriptors. These constitute a total of 209 numeric properties calculated from the atoms and connection table of the molecule. The RDKit and MOE descriptors were standardized using Scikit-learn’s standard binary nature (i.e., 1 s and 0 s) before passing to the machine learning models, while the fingerprints were used as is. Overall, models were built using three sets of descriptors: 197 RDKit descriptors, 209 MOE descriptors and Morgan fingerprints (1,024 bits).

### 2.5 Predictive models

#### 2.5.1 Random forest

Random Forest (RF) model is a classification and regression method based on an ensemble (or a forest) of multiple decision trees (Breiman, 2001). The large number of independent trees allows RF to make a prediction based on the majority of votes from individual trees. Each decision tree is built using a bootstrapped sample of data comprising a subset of the training features. The trained forest can then be used to predict data that was not seen before. RF calculations are considered computationally inexpensive and the method is relatively robust against overfitting. RFs have been popularly applied in development of machine learning models for predicting a range of drug discovery tasks and has been proven to perform on par with newer architectures such as deep neural networks and graph convolutional neural networks (Yang et al., 2019). In this study, we used “RandomForestClassifier” from Scikit-Learn, a Python framework for machine learning. Hyperparameter tuning was performed using the “GridSearchCV” method from Scikit-Learn.

#### 2.5.2 XGBoost

XGBoost stands for “eXtreme Gradient Boosting”. Although it is a classification and regression method based on an ensemble of trees, it is different from RF because it uses gradient boosting algorithm instead of bagging (Chen and Guestrin, 2016). Boosting allows the model to learn from errors after each round of boosting. XGBoost has been used in a variety of data science tasks and is known for speed and performance even when trained on large datasets as it supports distributed and parallel computing (Sheridan et al., 2020). In this study, we used the “XGBClassifier” method from XGBoost Python module. Similar to RF, hyperparameter tuning was performed using Scikit-Learn’s “GridSearchCV” method.

#### 2.5.3 Histogram gradient boosting

Histogram gradient boosting (HGB) is another ensemble classification and regression method that uses the boosting technique (Friedman, 2002; Ke et al., 2017). Compared to XGBoost, histogram gradient boosting is faster when dealing with large datasets. Since our project doesn’t have missing values, during training, the tree growers learn at each split point, and samples are mapped to whichever child has the most samples. We used the “HistGradientBoostingClassifier” module from Scikit-Learn Python package. Hyperparameter tuning was performed and class weights were applied when training the models, in a manner similar to RF and XGBoost models.

#### 2.5.4 Graph convolutional neural network (GCNN)

Due to the smaller size of the PAMPA-BBB permeability dataset as compared to our ADME Tier I endpoints (Siramshetty et al., 2021) (rat liver microsomal stability, PAMPA-GIT pH 7.4 permeability and kinetic aqueous solubility), we decided not to pursue building predictive models using deep neural networks. However, since GCNN demonstrated superior performance in predicting PAMPA permeability at pH 5 as compared to RF, XGBoost, and deep neural network methods (Williams et al., 2022), we decided to build a GCNN model for our PAMPA-BBB dataset. Briefly, the GCNN method takes chemical structures as input and transforms them into molecular graph representation where the nodes represent atoms and the edges represent bonds between the atoms. The network constructs a learned molecular representation by operating on graph structures in two phases: a message passing phase in which information is transmitted across the molecule and a read-out phase on which the learned representation is used to make predictions. In this study, we used Chemprop (https://github.com/chemprop/chemprop), a GCNN implementation in Python for prediction of molecular properties by retaining default parameters from the package.

### 2.6 Model evaluation

In order to identify the best performing model and validate its performance on a test set, we divided the original training set into an internal training set and an internal test set at 80:20 ratio, following a k-fold cross-validation scheme, and kept the validation set completely independent. The “train_test_split” method from Scikit-Learn was used to partition the training set for a total of five times and model performances were averaged over the five individual runs. For model evaluation, we used balanced accuracy (BACC) and AUC-ROC. BACC, calculated by averaging sensitivity and specificity, is an evaluation metric that is preferred when the dataset is imbalanced. Sensitivity is the probability of the true positive results, while specificity is the probability of the true negative results. AUC-ROC is the area under the ROC curve, where ROC stands for receiver operating characteristic curve which plots true positive rate (i.e., sensitivity) against false positive rate (i.e., 1-specificity). AUC-ROC estimates the ability of a model to distinguish between class 1 and class 0. The higher the value, the better the model is at separating class 1 and class 0. The model evaluation metrics have a numeric value between 0 and 1 and can be calculated using the four elements of confusion matrix, i.e., true positives (TP), false positives (FP), true negatives (TN), and false negatives (FN).

$$sensitivity=\frac{TP}{TP+FN}$$

$$specificity=\frac{TN}{TN+FP}$$

$$Balanced\;Accuracy\left(BACC\right)=\frac{sensitivity+specificity}{2}$$

TP = True Positive, FP = False Positives, TN = True Negatives, FN = False Negatives.

## 3 Results

### 3.1 Assay performance, description of dataset and distribution of molecular properties

Three control compounds, caffeine (low permeability), carbamazepine (moderate permeability), and progesterone (high permeability) were utilized in each plate for over 50 plates to provide evidence of assay quality. The minimum significant ratio (MSR) for all control compounds was below 3.5 as shown in Table 2, suggesting good assay reproducibility over a wide range of permeability values. Since all values for our low permeability control were below limit of quantification, S. D and MSR values were not calculated.

**TABLE 2:** Assay reproducibility data for control compounds, comprising mean and S.D of PAMPA-BBB permeability and the calculated minimum significant ratio (MSR) values. Since all values for caffeine were below limit of quantitation, S.D and MSR values were not calculated.

Compound	P_e_ (x 10^−6^ cm/sec)	MSR (10^2√2*S.D.^)
Caffeine	<1	N/A
Carbamazepine	21 ± 2.3	2.2
Progesterone	87 ± 10.1	3.4

**FIGURE 1:**
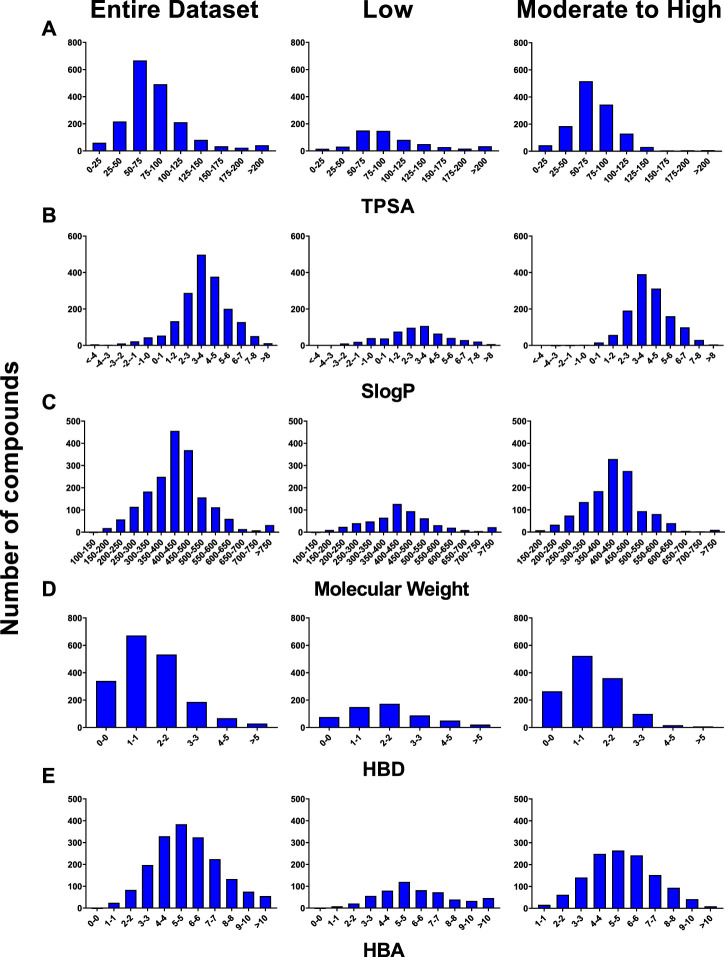
Distribution of the PAMPA-BBB dataset based on **(A)** TPSA, **(B)** SLogP, **(C)** Molecular Weight, **(D)** HBD, and **(E)** HBA.

The majority of compounds in our dataset (70% of total) fell into the moderate to high category, while only 30% of compounds were classified as low permeability compounds. Molecular properties, SLogP [RDKit toolkit’s open source implementation of Wildman and Crippen’s logP, known as SLogP (Wildman and Crippen, 1999)], total polar surface area (TPSA), molecular weight (MW), hydrogen bond donors (HBD) and hydrogen bond acceptors (HBA) were calculated as described previously (Williams et al., 2022) to observe any inherent trends in the dataset. A significant portion of compounds from both permeability categories exhibited molecular weights ranging from 250 to 650 g/mol, had Log *p* values between 2 and 6, 0–2 HBD, 3-8 HBA, and possessed TPSA values below 100 (Figure 1). Interestingly, compounds with a moderate to high BBB permeability were observed to have higher SLogP values whereas, these compounds tended to have lower HBD and TPSA. No significant trends were observed with regard to HBA or MW.

### 3.2 Correlation between PAMPA-GIT and PAMPA-BBB

**FIGURE 2:**
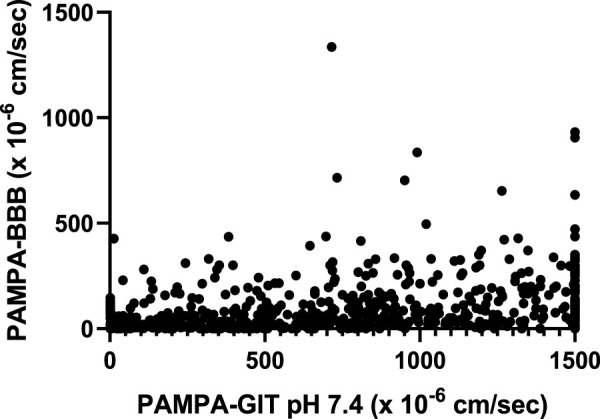
Correlating Tier I PAMPA-GIT pH 7.4 and PAMPA-BBB data.

NCATS utilizes high-throughput PAMPA assays pH 5 and pH 7.4 (PAMPA-GIT) for predicting gastrointestinal permeability (Sun et al., 2017; Williams et al., 2022). These assays are routinely employed as a component of the Tier I ADME assay suite. We attempted to establish correlations by comparing the Tier I PAMPA-GIT and PAMPA-BBB data to identify any visible trends. As the count of shared compounds between PAMPA-GIT pH 7.4 and PAMPA-BBB (750 compounds) were notably higher in contrast to the shared compounds between PAMPA-GIT pH 5 and PAMPA-BBB (37 compounds), we focused exclusively on conducting correlations for the former set. No discernable linear (Figure 2) or categorical/rank ordering (data not shown) trends were observed.

### 3.3 Correlating *in vitro* PAMPA-BBB data with *in vivo* B/P ratios

**FIGURE 3:**
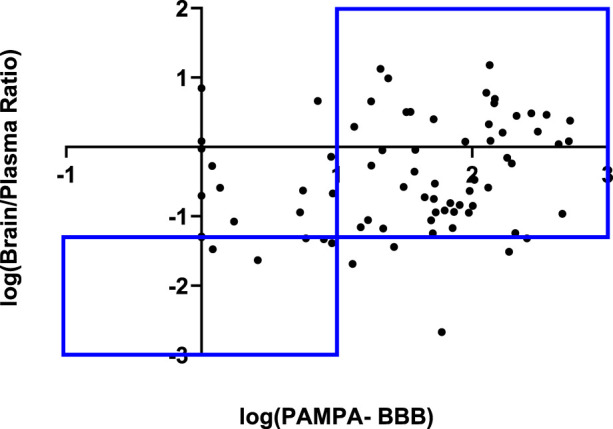
Categorical correlation of *in vitro* PAMPA-BBB permeability versus *in vivo* Brain/Plasma Ratio.

To underscore the significance of the PAMPA-BBB assay, we correlated log PAMPA-BBB values with *in vivo* B/P ratios. From our in-house pharmacokinetic database, which primarily consists of studies conducted in mice (90%) and rats (10%), we extracted a subset of 74 compounds that had available B/P ratios. While no linear correlation was observed, a 77% categorical correlation using B/P and PAMPA-BBB cut-off values at 5% and 10 × 10^−6^ cm/sec respectively was identified (Figure 3). In addition to passive diffusion, other major mechanisms of transport into the brain involve paracellular transport, carrier mediated transport and receptor mediated transport. The paracellular transport routes primarily pertain to the movement of small hydrophilic compounds (Barar et al., 2016). Carrier mediated transport is primarily responsible for the transportation of glucose, amino acids, nucleic acids, ions, prostaglandins, and various other small polar molecules. While more than 20 carrier mediated transporters have been identified, the major ones include glucose transporter 1 (GLUT1), monocarboxylate transporters 1/2 (MCT1/2), L-system neutral amino acid transporter 1 (LAT1) (Saunders et al., 2013; Sweeney et al., 2019) and organic anion transporting polypeptides (OATP) 1A2/OATP2B1 (Roth et al., 2012). Receptor-mediated transport serves as the primary mechanism for the transportation of larger molecules such as peptides, proteins, and lipids (Yang et al., 2020). Despite the limitation of the PAMPA-BBB assay in modeling these transport mechanisms, we observed a remarkably strong categorical correlation. It is well known that efflux, primarily by P-glycoprotein (Pgp) is a major barrier for xenobiotic compounds to penetrate the BBB. To understand if a better correlation could be observed between PAMPA-BBB and B/P ratios, we tested 74 compounds from our *in vivo* dataset in our in-house MDCK-MDR1 assays, with the aim of identifying potential Pgp substrates. While 14 compounds were identified to have moderate efflux ratios, i.e., efflux ratios >5 and 4 compounds were identified to have high efflux ratios, i.e., efflux ratios >20, no significant change in the categorical correlation was observed after removal of these compounds (data not shown).

Breast Cancer Resistance Protein (BCRP) is another extensively recognized efflux transporter, and recent research has indicated that while its expression in rodents is 2–3 folds lower than in non-human primates and humans, its expression in the human brain surpasses that of Pgp (Feng et al., 2018). Many isoforms of Multidrug Resistance-Associated Proteins (MRPs) have been found to be expressed on the BBB and are believed to play a significant part in BBB transport (Zhang et al., 2000; Zhang et al., 2022). Hence, even though we took into account the influence of the primary efflux transporter at the rodent blood-brain barrier (Pgp), it did not exhibit a substantial effect in our *in vitro-in vivo* correlation. Nonetheless, different transporters might exhibit diverse levels of influence on this correlation.

### 3.4 Performance of prediction models

**FIGURE 4:**
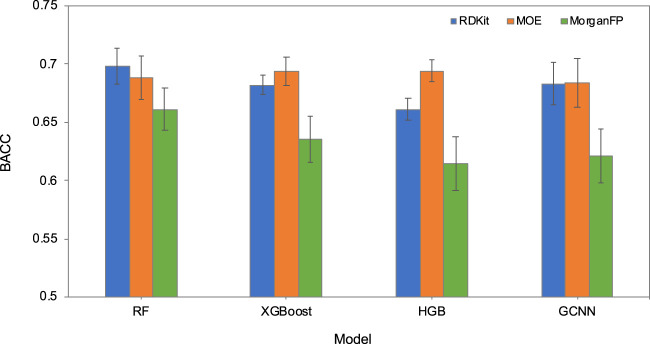
Cross-validation performance (balanced accuracy, BACC) of RF, XGBoost, HGB, and GCNN models, each based on RDKit descriptors, MOE descriptors and Morgan fingerprints.

The ability of machine learning methods to learn the prediction task was first evaluated in a five-fold cross-validation (5-CV) based on the internal training and test sets. Figure 4; Supplementary Table S1 show the 5-CV performance of the baseline models. Among the 12 models based on four different methods and three different chemical descriptors, RF based on RDKit descriptors performed the best, followed by XGBoost and HGB models based on MOE descriptors. The models based on Morgan fingerprints consistently performed poorly compared to the models based on RDKit and MOE descriptors. Due to the superior performance of RDKit descriptors in 5-CV, we evaluated Chemprop’s GCNN method by using RDKit and MOE descriptors as additional features and compared with the RF models based on RDKit and MOE descriptors. To identify the most suitable model, all four models underwent validation using the validation set, and the model exhibiting the highest balanced accuracy on this validation set would be selected as the most optimal. Results identified the GCNN model with RDKit descriptors as the best performing model (Table 3). The complete validation set results can be found in Supplementary Table S2.

**TABLE 3:** Performance of RF and GCNN models using RDKit and MOE descriptors in cross-validation and on the validation set.

Models	Descriptors	5-fold CV BACC	Validation set BACC
Random forest	RDKit	0.698 ± 0.015	0.708
	MOE	0.688 ± 0.019	0.659
GCNN	RDKit	0.683 ± 0.018	0.723
	MOE	0.684 ± 0.021	0.693

Due to the lack of publicly accessible PAMPA-BBB data, from both literature and compound bioactivity databases such as ChEMBL, we were unable to evaluate our models on completely unseen data. However, our validation set was derived from most recent drug discovery projects, which mimics a practical real-time scenario. Several compounds from recent projects belong to newer chemical spaces explored by medicinal chemists in the pursuit of novel drugs. However, this observation may not be applicable to all validation set compounds as can be seen in the chemical space distribution (Supplementary Figure S1).

### 3.5 Boiled-Egg to predict PAMPA-BBB permeability

Lipophilicity and polarity are two physicochemical properties that are relevant for absorption of small molecules through biological membranes. Egan et al. (2000), developed a descriptive representation using these two properties in order to distinguish well-absorbed and poorly absorbed compounds. Lipophilicity and polarity were described using n-octanol/water partition coefficient [ALOGP98; which is an implementation of LogP, originally proposed by Ghose and Crippen (Ghose and Crippen, 1986)] and TPSA, respectively. When the two computed properties were plotted against each other, a favorable region for gastrointestinal absorption delineated, and as the region populated with most of the well-absorbed molecules was elliptical in shape, it was called Egan Egg. Unlike rule-based models and machine learning models, this representation not only provides thresholds for lipophilicity and polarity but also an estimate of how far a molecule is from the favorable region. In a 2016 study, Daina and Zoete extended Egan’s Egg concept by amending the methodological aspects and assessed the predictive power of the model (Daina and Zoete, 2016). In addition to predicting gastrointestinal absorption, this model also predicts the brain permeability of small molecules by passive diffusion. A BOILED-Egg (Brain Or IntestinaL EstimateD permeation predictive model) was constructed by plotting Wildman and Crippen log P (WLOGP) (Wildman and Crippen, 1999) against TPSA for: 1) a total of 660 molecules with human intestinal absorption data collected from literature, patents, and other databases, and 2) a total of 260 molecules with brain permeability data. From both plots, they identified the best elliptical regions with the highest number of well-absorbed and permeable molecules which were combined to yield the BOILED-Egg predictive model. Molecules that fall within the white region are those with highest probability to be absorbed in the gastrointestinal tract and those that fall within the yellow region (yolk) are those with highest probability to permeate into brain. The BOILED-Egg model was implemented and made publicly available on the Swiss-ADME prediction portal.

**FIGURE 5:**
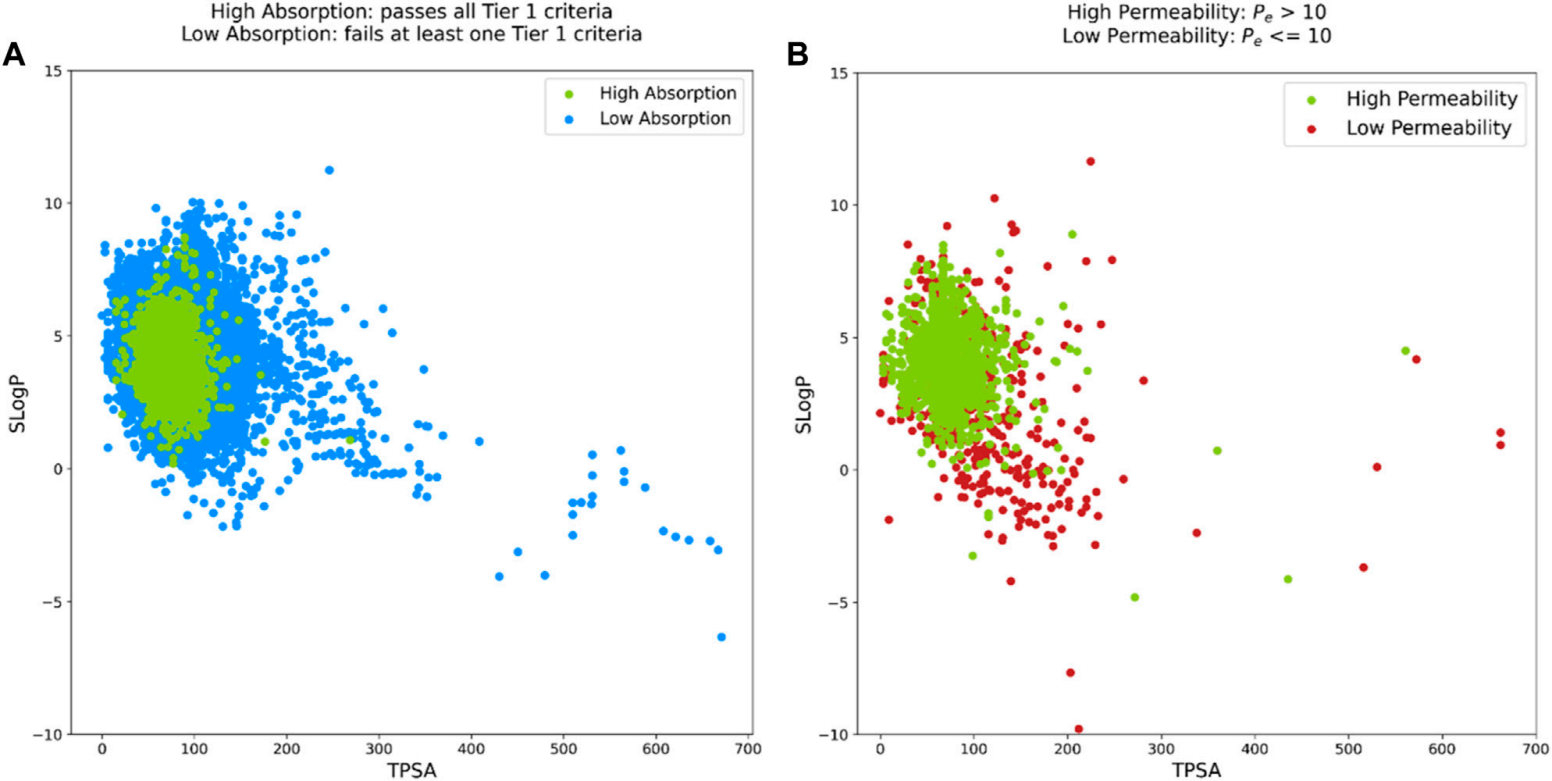
Overview of BOILED-Egg like analysis based on two distributions: **(A)**. SLogP versus TPSA for Tier I dataset where green dots are “High Absorption” molecules and blue dots are “Low Absorption” molecules. **(B)** SLogP versus TPSA for PAMPA-BBB dataset where green dots are “High Permeability” molecules and brick red dots are “Low Permeability” molecules.

We aimed to evaluate this approach using our internal data. For brain penetration, we used all molecules from the PAMPA-BBB dataset. As our Tier I PAMPA permeability might not completely translate to intestinal absorption of small molecules, we combined data from all our Tier I endpoints (rat liver microsomal stability, PAMPA-GIT permeability, and kinetic aqueous solubility) as a loose approximation for intestinal absorption/bioavailability. In the final dataset comprising a total of 18,461 molecules, a molecule was assigned to the “High Absorption” class if it passes all Tier I criteria, i.e., high liver microsomal stability, high PAMPA permeability and high kinetic aqueous solubility. If a molecule did not satisfy any of these three criteria, it was assigned to the “Low Absorption” class. Another slight methodological modification was to use SLogP instead of WLogP as the latter was not available in the commonly used open-source molecular descriptor calculation tools. Figures 5A, B represent the SLogP versus TPSA distributions for the two datasets. Although the upper and lower thresholds vary and the “High Permeability” compounds do not fall within the boundaries of the “High Absorption” region, the respective elliptical distributions obtained with our internal data closely match with those from the BOILED-Egg model. It must be noted that in their original work, Daina and Zoete minimized the number of poorly absorbed molecules in the human intestinal absorption data which is a reason for overrepresentation of the well absorbed compounds leading to the formation of white region of the boiled egg. This analysis suggests that it might not be straightforward to develop a BOILED-Egg like model for predicting PAMPA-BBB permeability.

## 4 Discussion

Owing to its many advantages including high-throughput nature, speed, and low cost, PAMPA-BBB assay is routinely used for rank ordering compounds in drug discovery. Using a combination of basic and advanced machine learning techniques, we aimed to develop and validate a QSAR model for predicting passive BBB permeability using our in-house ∼2,000 compound PAMPA-BBB dataset. Out of the multiple methods analyzed, GCNN exhibited the highest validation set accuracies >72%. The best model along with a subset of our PAMPA-BBB dataset has been made publicly available to benefit the drug discovery community.

By comparing *in vitro* PAMPA-BBB data with *in vivo* B/P ratios, we identified a categorical correlation of 77%, emphasizing the usefulness of this assay. Despite its inability to simulate active transport, the PAMPA-BBB assay was able to achieve a significant correlation with *in vivo* results. The presence of efflux transporters, primarily Pgp, at the BBB is widely recognized as a substantial hurdle for drug penetration into the brain. To understand the impact of Pgp efflux on compound brain penetration, we tested 74 compounds from our *in vivo* dataset in MDCK-MDR1 assays and found only 4 compounds with high efflux ratios. The lack of compounds with high efflux in our dataset could be one of the factors as to why we achieved such a strong correlation between PAMPA-BBB permeability and B/P ratios in our dataset. We plan to routinely monitor these correlations as our *in vivo* datasets expand. In addition, we performed a correlation analysis of 750 overlapping compounds from our PAMPA-BBB dataset and our Tier I PAMPA-GIT pH 7.4 dataset. No linear or categorical correlation was observed indicating their individual assay relevance.

Due to the uneven distribution of compounds between the two classes, RF, XGBoost and HGB models were trained using class weights. We attempted to increase the model performance by generating a more balanced dataset using a different cutoff value for BBB permeability. When PAMPA-BBB permeability cutoff of 40 × 10^−6^ cm/sec was used as the threshold to classify compounds, the balanced accuracy for the training set (5-CV) and validation set increased to 75% and 74% respectively. Moreover, the AUC-ROC increased to 81% for both datasets. In addition to GCNN, simple decision trees, logistic regression and multi-layer perceptron models were also built and compared with the already developed models, however, none of them performed better than the GCNN model based on RDKit descriptors. Dataset size and limited coverage of chemical space can be a confounding factor in modeling and can cause the model to have low predictive performance. Despite this, the GCNN model based on the default parameters performed well compared to other classifiers. On the other hand, sparse molecular descriptors such as molecular fingerprints did not provide competitive predictive performance when compared to whole molecular properties like RDKit descriptors. They are limited by their inefficiency in projecting complex multidimensional objects such as molecules onto a single dimension (i.e., a bis string representation) where there is no meaningful relationship between two bits that are next to each other (Feinberg et al., 2020). Although the differences in cross-validation balanced accuracy values between GCNN and RF were not significant, GCNN performed better on the validation set compounds.

Concurrently, we assessed feature importance using the RF model based on RDKit descriptors and identified the top five importance features: partition coefficient, octanol/water partition coefficient, total polar surface area (TPSA), quantitative estimation of drug-likeness (qed), and van der Waals surface area Estate 5 (VSA_Estate5). The pH-partition hypothesis states that only uncharged molecules can permeate through lipophilic membranes (Shore et al., 1957; Williams et al., 2022) and that permeability of a molecule would be the greatest when it is least charged, making ionization status a very important factor in determining compound permeability. This is substantiated by the observation that various descriptors employed in the construction of our model, such as partition coefficient, octanol/water partition coefficient, molecular charge, electronegativity, atomic charges, bond type, and molecular connectivity, are either directly or indirectly related to ionization. In our analysis of the dataset, we demonstrated that, in contrast to acidic compounds, basic compounds exhibited higher PAMPA-BBB permeability values (Supplementary Figure S2). Consequently, it is not surprising that partition coefficient and octanol/water partition coefficient; two properties that rely heavily on ionization state of the molecule rank high in the feature importance list. It is interesting to see SLogP and TPSA as important features since both properties show trends in the physicochemical property distribution graphs in Figure 1.

Several models that predict BBB permeability have been reported in literature. Wang et al. (2015) summarized several of these efforts (up to 2016) in their work where they compiled a total of 439 compounds from multiple resources to develop a consensus QSAR model. Although they started developing models using MOE descriptors alone, they demonstrated that combining them with biological descriptors improved model performance. In this case, biological descriptors including bioassays on membrane transporters were extracted from PubChem. Due to the direct dependence of small molecules on these transporters to pass through the BBB, the transporter activities served as biological descriptors and thereby helped improve predictive performance. We further extended the list to include the most recent studies that reported models based on both classical machine learning methods (RF, SVM, etc.) and neural network-based methods. Supplementary Table S3 in the supporting information provides a summary of these studies in addition to those summarized by Wang et al. (2015). Majority of datasets from prior publications typically encompass around 400 compounds within their training sets, whereas we possess a comparably larger dataset of approximately 2,000 compounds. An exception to this is the dataset compiled by Shaker et al. (2021) which comprises a total of 7,162 compounds collected from several literature reports and data repositories. Shaker et al. (2021), reported a classification model based on light gradient boosting machine algorithm that provided an accuracy of 89% on a test set derived from the same dataset. While our dataset is smaller than the dataset from Shaker et al. (2021), we would like to highlight that our dataset was generated using the same protocol, at a single laboratory and is based on a high throughput PAMPA-BBB assay. Although we could only disseminate a subset of this dataset, our best model based on the full dataset is publicly available on the NCATS Open Data ADME portal.

It has been widely recognized that unbound drug concentration at the site of action is the main driver for eliciting a pharmacological response (Kalvass and Maurer, 2002; Kalvass et al., 2007; Watson et al., 2009). Unbound B/P partition coefficient or K_p,uu,brain_ describes the unbound drug concentrations in brain relative to plasma at equilibrium. Some of the mainstream methods for K_p,uu,brain_ determination include microdialysis sampling (Ooie et al., 1997; Friden et al., 2007), brain slice assays (Friden et al., 2007; Friden et al., 2009) and equilibrium dialysis using brain tissue homogenates (Friden et al., 2007; Wan et al., 2007; Liu et al., 2009). Since microdialysis and brain slice assays are complicated and costly, equilibrium dialysis method using brain homogenate is the most common method used in the pharmaceutical industry (Wan et al., 2007; Watson et al., 2009). A recent survey among scientists from 14 top pharmaceutical companies was conducted with the aim of understanding how K_p,uu,brain_ values are utilized in their respective companies. The researchers strive to establish a correlation between the measured K_p,uu,brain_ and *in vitro* assays such as MDCK-MDR1 and MDCK-BCRP efflux assays, with the aim of utilizing these *in vitro* assays to screen and prioritize compounds (Loryan et al., 2022). Although our *in vivo* PK dataset is comprised of drugs encompassing diverse therapeutic areas, only a small fraction of compounds target the CNS. However, with the advent of the NIH Helping to End Addiction Long-term (HEAL) initiative (https://heal.nih.gov), there has been an influx of projects at NCATS that target the CNS and we plan to develop correlations between experimental K_p,uu,brain_ values and our in-house PAMPA-BBB assays.

## 5 Conclusion

In conclusion, our research has yielded successful predictive models using by far the most extensive PAMPA-BBB dataset, with the highest-performing model now accessible on our Open Data ADME portal. Through a comprehensive evaluation, we compared state-of-the-art machine learning methods with recent architectures like graph neural networks, utilizing diverse molecular descriptors as features. Notably, a subset of our compiled PAMPA-BBB dataset has been shared as a PubChem bioassay record (AID: 1845228). Moreover, we identified a significant 77% correlation between our *in vitro* PAMPA-BBB data and *in vivo* brain permeation data, highlighting the considerable promise inherent in the PAMPA-BBB assay and the models derived from this data for evaluating the BBB permeability of small molecules. This strong correlation will instill confidence among medicinal chemists in applying these models to efficiently prioritize compounds for preclinical testing. By providing valuable insights into blood-brain barrier permeation, our research contributes significantly to advancing drug discovery efforts. Ultimately, the successful implementation of these *in silico* tools holds the promise of revolutionizing the drug discovery process, leading to considerable time savings and improved efficiency.

## Data Availability

The datasets presented in this study can be found in online repositories. The names of the repository/repositories and accession number(s) can be found below: PubChem bioassay record (AID: 1845228).
