# Supplementary material for: Development and validation of PAMPA-BBB QSAR model to predict brain penetration potential of novel drug candidates
Source: Front Pharmacol. 2023 Dec 1;14:1291246. doi: 10.3389/fphar.2023.1291246 (PMC10722238; doi:10.3389/fphar.2023.1291246)

Development and Validation of PAMPA-BBB QSAR Model to Predict Brain Penetration Potential of Novel Drug Candidates

***Rintaro Kato^1*^, Wenyu Zeng^1*^***, Vishal B. Siramshetty^1^, Jordan Williams^1,2^, Md Kabir^1,3^, Natalie Hagen^1,4^, Elias C. Padilha^1^, Amy Q. Wang^1^, Ewy A Mathé^1^, Xin Xu^1^ & Pranav Shah^1^

1: National Center for Advancing Translational Sciences (NCATS), 9800 Medical Center Drive, Rockville, Maryland 20850, United States

2: Current Affiliation: Perelman School of Medicine. Pharmacology Graduate Group, University of Pennsylvania - Philadelphia, PA 19104, United States

3: Current Affiliation: The Graduate School of Biomedical Sciences, Department of Pharmacological Sciences, Icahn School of Medicine at Mount Sinai, New York, New York 10029, United States.

4: Current Affiliation: Perelman School of Medicine, University of Pennsylvania – Cell and Molecular Biology Group: Microbiology, Virology, and Parasitology Program, Philadelphia, PA 19104, United States

*Authors made equal contributions

Corresponding Author: Pranav Shah, pranav.shah@nih.gov

**Figure S1**. Chemical space distribution of the training set (blue) and external set compounds (orange) using the dimension reduction techniques: (A) principal component analysis (PCA); (B) t-distributed stochastic neighbor embedding (t-SNE). Scaled RDKit descriptors (n=197) were used as high-dimensional input data. In both plots, the first two components of the low-dimensional data were visualized.

**Figure S2.** PAMPA-BBB permeability distribution of our dataset by ion class.


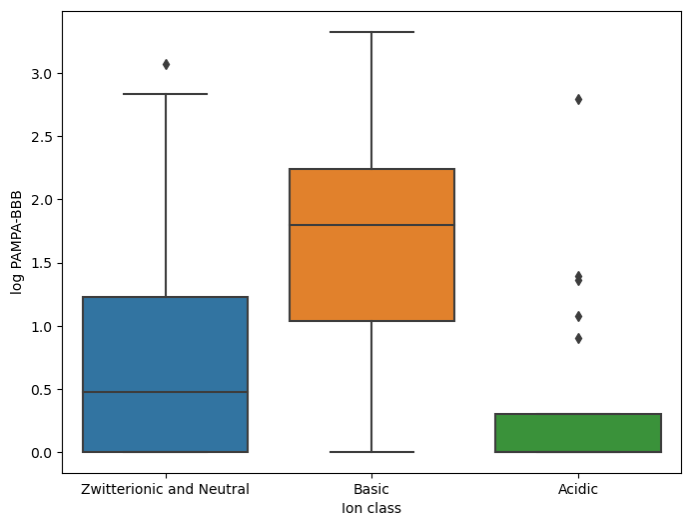

Supplement: Supplementary file 1 [file DataSheet2.docx]
